# Supplementary material for: Real World Evaluation of the Prosigna/PAM50 Test in a Node-Negative Postmenopausal Swedish Population: A Multicenter Study
Source: Cancers (Basel). 2022 May 25;14(11):2615. doi: 10.3390/cancers14112615 (PMC9179899; doi:10.3390/cancers14112615)
Supplement: Supplementary file 1 [file cancers-14-02615-s001.zip › cancers-1684845-supplementary.pptx]

## Slide 1
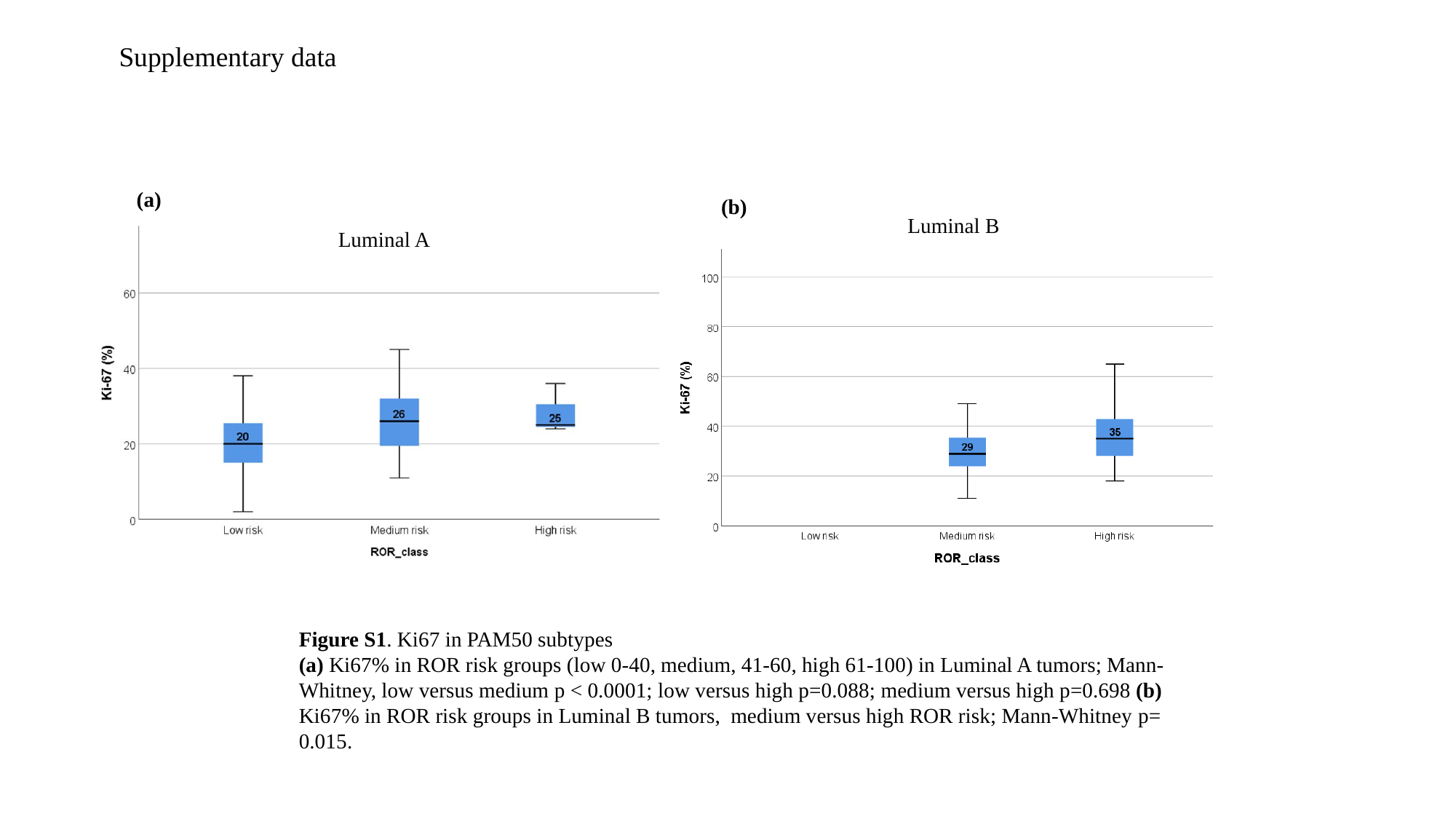

Supplementary data
(a)
(b)
Luminal B
Luminal A
Figure S1. Ki67 in PAM50 subtypes
(a) Ki67% in ROR risk groups (low 0-40, medium, 41-60, high 61-100) in Luminal A tumors; Mann-Whitney, low versus medium p < 0.0001; low versus high p=0.088; medium versus high p=0.698 (b) Ki67% in ROR risk groups in Luminal B tumors, medium versus high ROR risk; Mann-Whitney p= 0.015.

## Slide 2
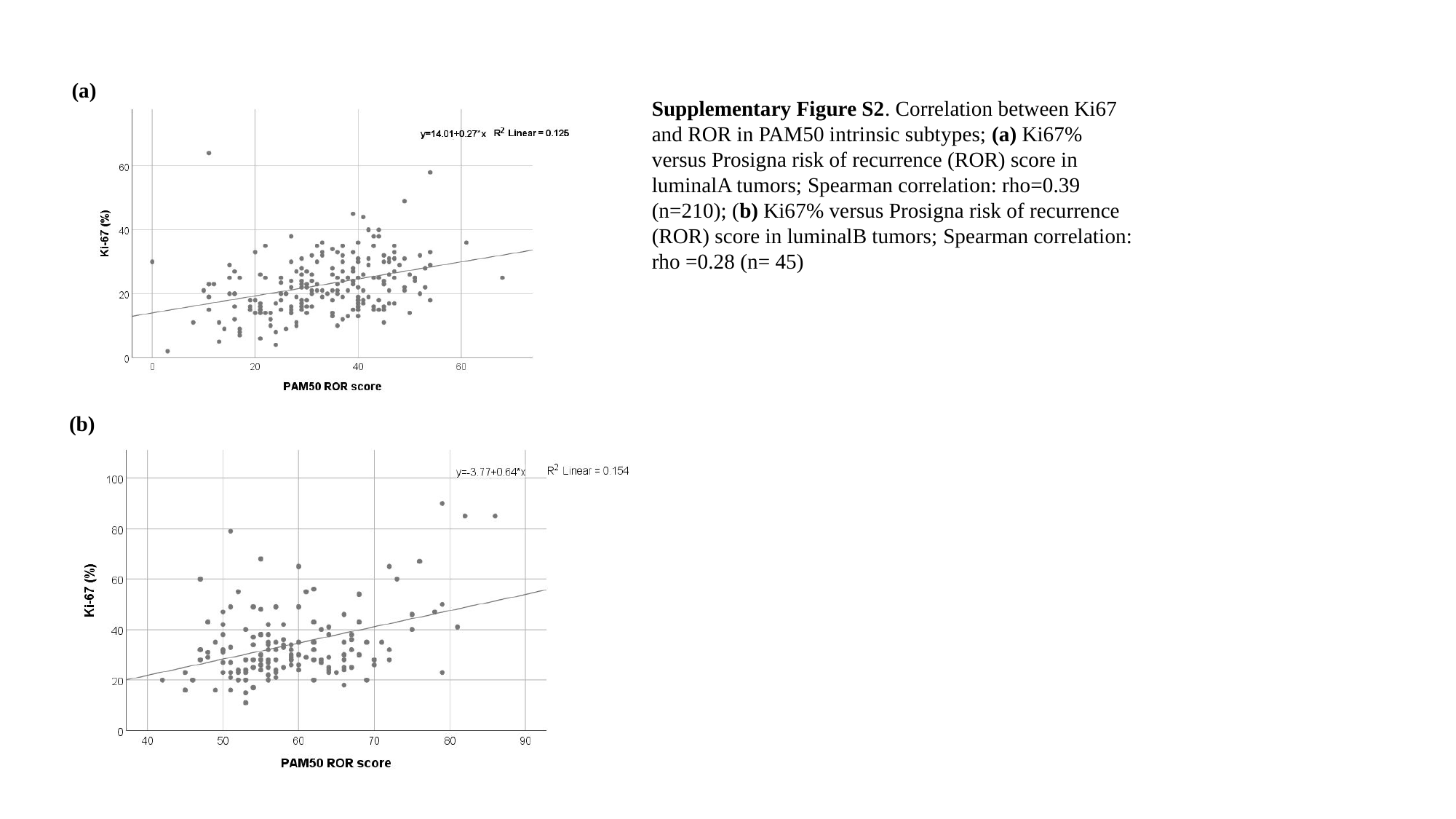

(a)
Supplementary Figure S2. Correlation between Ki67 and ROR in PAM50 intrinsic subtypes; (a) Ki67% versus Prosigna risk of recurrence (ROR) score in luminalA tumors; Spearman correlation: rho=0.39 (n=210); (b) Ki67% versus Prosigna risk of recurrence (ROR) score in luminalB tumors; Spearman correlation: rho =0.28 (n= 45)
(b)

## Slide 3
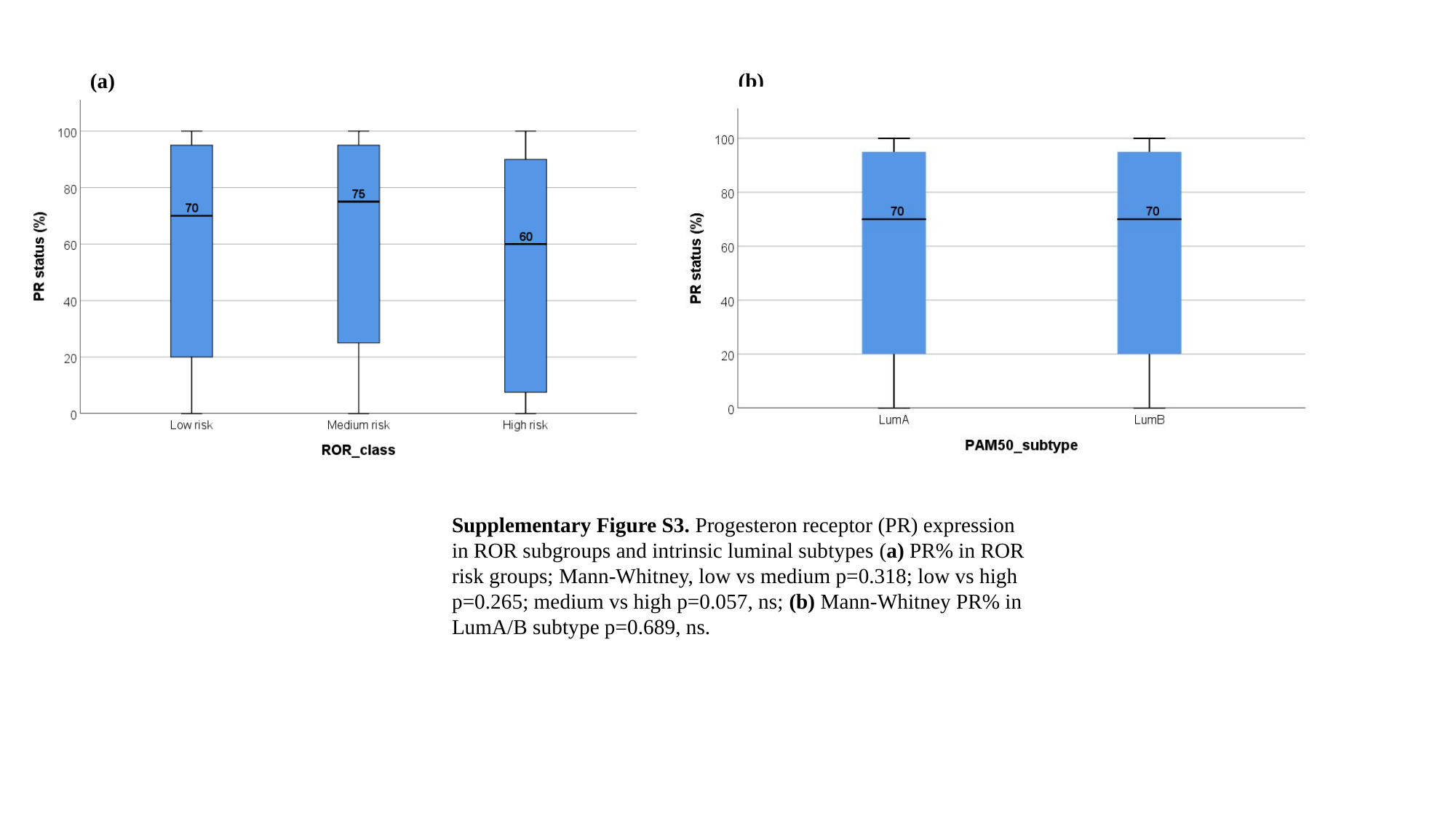

(b)
(a)
Supplementary Figure S3. Progesteron receptor (PR) expression in ROR subgroups and intrinsic luminal subtypes (a) PR% in ROR risk groups; Mann-Whitney, low vs medium p=0.318; low vs high p=0.265; medium vs high p=0.057, ns; (b) Mann-Whitney PR% in LumA/B subtype p=0.689, ns.

## Slide 4
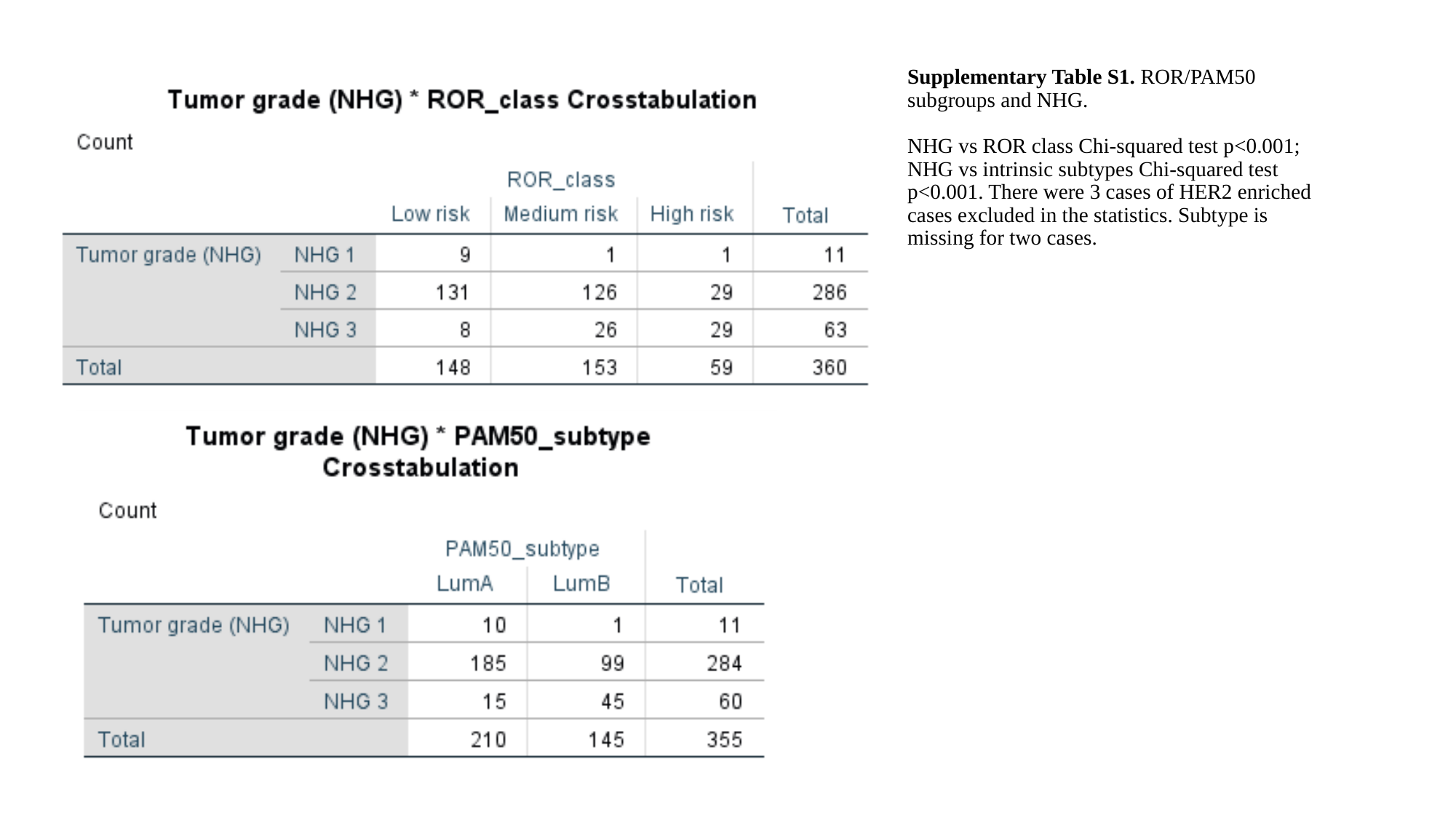

# Supplementary Table S1. ROR/PAM50 subgroups and NHG. NHG vs ROR class Chi-squared test p<0.001; NHG vs intrinsic subtypes Chi-squared test p<0.001. There were 3 cases of HER2 enriched cases excluded in the statistics. Subtype is missing for two cases.

## Slide 5
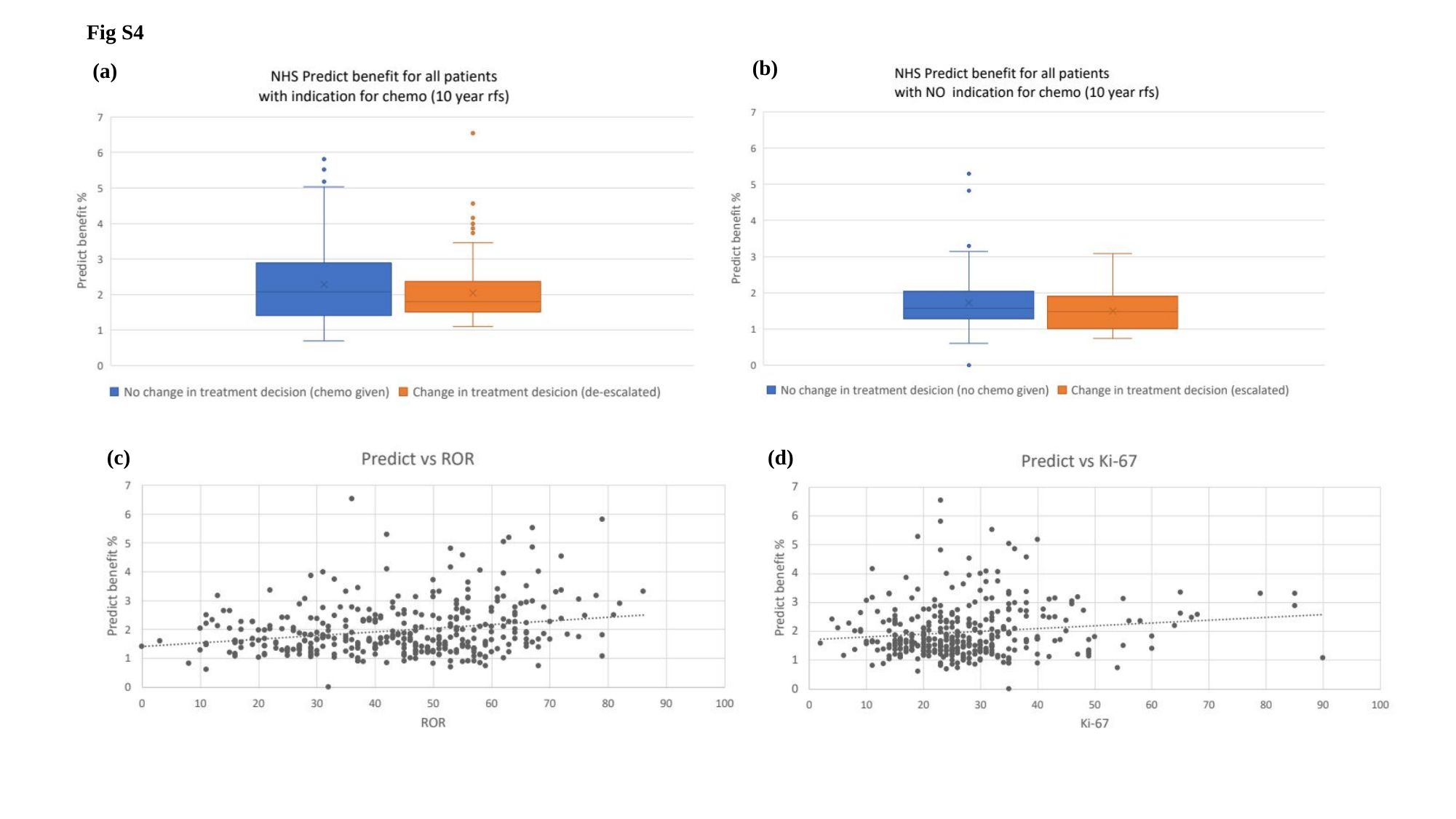

Fig S4
(b)
(a)
(d)
(c)

## Slide 6
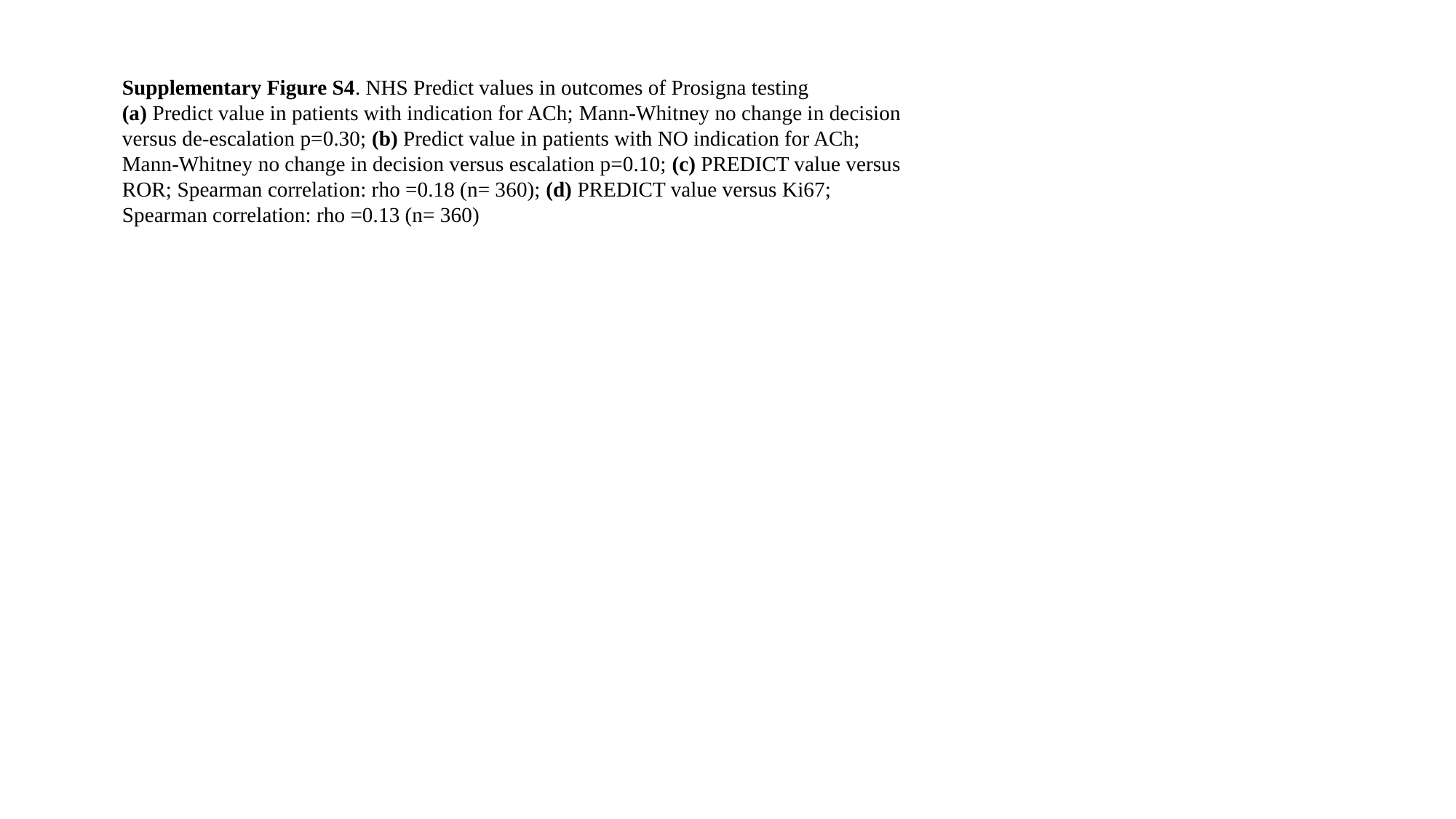

Supplementary Figure S4. NHS Predict values in outcomes of Prosigna testing
(a) Predict value in patients with indication for ACh; Mann-Whitney no change in decision versus de-escalation p=0.30; (b) Predict value in patients with NO indication for ACh; Mann-Whitney no change in decision versus escalation p=0.10; (c) PREDICT value versus ROR; Spearman correlation: rho =0.18 (n= 360); (d) PREDICT value versus Ki67; Spearman correlation: rho =0.13 (n= 360)
